# Surgical management of chronic osteomyelitis with benign osteopetrosis: A case report

**DOI:** 10.1016/j.amsu.2021.102296

**Published:** 2021-04-20

**Authors:** Mohamed Raiteb, Sanaa Elmrini, Faiçal Slimani

**Affiliations:** a Oral and Maxillofacial Surgery Department, CHU Ibn Rochd, B.P 2698, Casablanca, Morocco; b Faculty of Medicine and Pharmacy, Hassan II University of Casablanca, B.P 5696, Casablanca, Morocco

**Keywords:** Osteopetrosis, Osteomyelitis, Mandibular, Case report

## Abstract

**Introduction:**

Osteopetrosis is a rare genetic bone disease caused by a functional abnormality of the osteoclasts. Until now there is no codified management for the complications of this pathology and few cases cited in the literature.

**Presentation of case:**

a 19-year-old adult followed in our maxillofacial surgery department in the IBN ROCHD University Hospital for chronic osteomyelitis complicating mandibular osteopetrosis with skin fistulas. Patient operated several times. The persistence of osteomyelitis prevents the installation of a dental prosthesis and the appearance of new fistulas with continuous flow of pus alters the patient's quality of life.

**Discussion:**

Osteopetrosis is a group of rare genetic diseases characterized by osteoclastic insufficiency, poor bone remodeling and increased bone density. the benign form of osteopetrosis called Albers-Schönberg disease. It is a genetically inherited autosomal dominant disease.

The large number of surgical interventions and the use of antibiotics for long periods of time (risk of development of resistance) significantly reduces the quality of life of patients. We must seek other measures to improve the prognosis and codify management.

**Conclusion:**

In osteopetrosis, the maxillofacial surgeons should be aware about the early diagnosis and the appropriate management of the signs and prevent complications.

## Highlights

• Osteopetrosis is a rare genetic bone disease.
• No codified management for the complications of this pathology.
• The maxillofacial surgeon must always choose between surgery and its risks and medical treatment.

## 1 Introduction

**Fig. 1:**
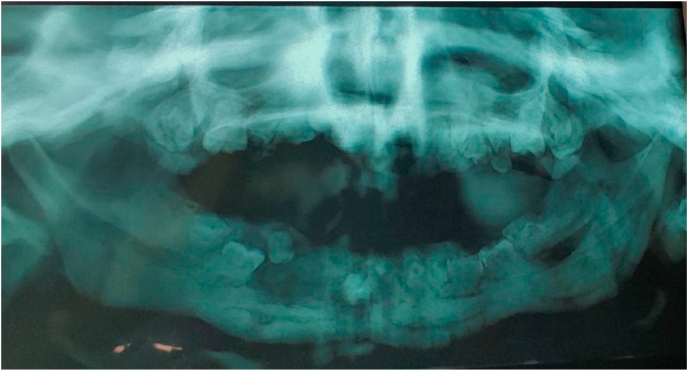
Panoramic radiograph showing the presence of osteomyelitis foci in the mandible and ectopic teeth.

**Fig. 2:**
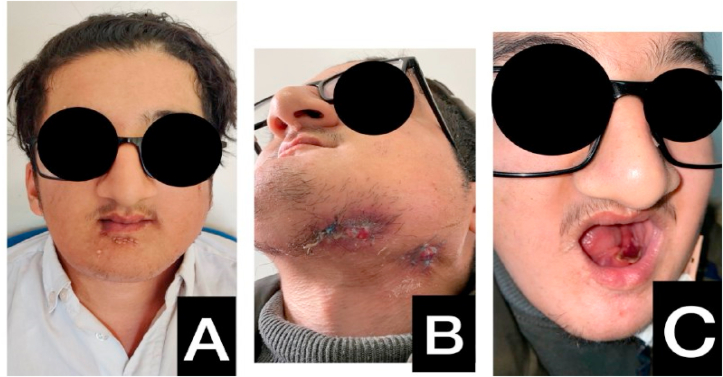
**A:** Preoperative clinical view of face **B:** persistence of two skin fistulas one month after the second surgery **C:** denudation of the left side of the mandibular bone one month after the second surgery.

Osteopetrosis is a rare genetic bone disease caused by a functional abnormality of the osteoclasts. Its incidence is 1 in 250,000 births [1]. It is described as an infantile malignant form of autosomal recessive transmission and a benign form called autosomal dominant Albers-Schönberg disease [2]. The diagnosis is often made following a complication or following a radiological examination. (see Fig. 1, Fig. 2)

Benign osteopetrosis is clinically manifested by fractures and osteomyelitis of the mandible [3] Osteomyelitis is a complication that can be serious and difficult to treat. At present, the curative treatment for malignant osteopetrosis is allogenic hemopoietic stem cell transplantation [4]; however, only supportive care is available for benign osteopetrosis [5]. Therefore, symptom management of benign osteopetrosis is important to increase the patient survival rates [6]. We report the case of an adult who was followed for osteopetrosis since childhood and who developed mandibular osteomyelitis. This case report has been reported in line with the SCARE Criteria [7].

This case report has been reported in line with the SCARE Criteria [8].

## 2 Case report

Patient aged 19 years old, male, university student and resident in central Morocco. He presented for 3 years a painful swelling on the left side a few days after the installation of a dental prosthesis, the patient was put under amoxicillin by his dentist without improvement, the evolution was marked by the appearance of a fistula facing the chin with pus. Faced with no improvement, the dentist referred him to us for further treatment.

The patient has been followed for osteopetrosis since childhood with a history of two fractures of the right and left femur following a minor trauma that was treated orthopedically.

No notion of similar cases in the family and no notion of consanguinity. No notion of allergy.

On admission to hospital, clinical examination revealed the presence of pus in the endobuccal area with chin fistula. The biological examination revealed a hemoglobin level of 12 g/l, the rate of GB 12000/mm. The panoramic X-ray showed persistent dental germs with foci of mandibular osteitis. 5 days later, the surgical intervention was performed by the chief professor of our department who has 16 years of experience, the patient under general anesthesia was given a sequestrectomy with extraction of 3 dental germs and was put under intravenous antibiotic therapy based on amoxicillin - clavulanic acid for 6 days and then relayed orally for 15 days. The pathological examination confirmed the diagnosis of osteomyelitis.

The patient was lost to follow-up for 6 months and was then seen at the clinic, where 3 skin fistulas with pus discharge were discovered, and the presence of mandibular bone sequestres visible on endobuccal examination.

The patient was depressed due to the continuous pus flow in the fistulas and therefore the absence of dentures (due to the persistence of mandibular osteomyelitis) prevented him from continuing his university studies. After a multidisciplinary team of dentists and psychiatrists, the decision was made to perform a second surgery with antidepressant treatment.

He again underwent a sequestrectomy with extraction of 4 teeth with ectopic aspects and curettage of the surrounding bone which appeared to be necrotic, then the 3 skin fistulas were removed and an attempt was made to cover the mandibular bone. The patient was put on ciprofloxacin (1g per day in two doses) intravenously for 5 days and then relayed orally.

The patient was seen at points J1, J7, J14 and J28 where two persistent fistulas were observed with denudation on the left side of the mandibular bone, a new bacteriological sample was taken and the patient is still on Ciprofloxacin.

## 3 Disscusion

Osteopetrosis is a group of rare genetic diseases characterized by osteoclastic insufficiency, poor bone remodeling and increased bone density [9].

No racial predilection was reported. However, inbreeding appears to be a contributing factor [10].

Early osteopetrosis is known as a severe autosomal recessive malignant childhood disease, usually fatal as a result of anemia with congestive heart failure or sepsis due to bone overgrowth in the bone marrow space. In adults, the same disease is known as late-onset benign ADO with a lower mortality rate.

The diagnosis of autosomal dominant osteopetrosis OAD is often made following a complication or following a radiological workup.

Examination of the oral cavity in patients with benign osteopetrosis objectively results in delayed eruption and impaction of teeth, malformed non-eruptive teeth, and early tooth loss [11].

Dental extractions may be necessary due to poor oral hygiene and the above-mentioned dental abnormalities. However, their completion is difficult and usually followed by extensive bone loss leading to prolonged osteomyelitis and fistulas. Even erupting teeth can lead to serious infection such as orbital cellulitis.

Currently, the curative treatment for malignant osteopetrosis is allogeneic hematopoietic stem cell transplantation [12]; however, only supportive care is available for benign osteopetrosis. Therefore, symptom management of benign osteopetrosis is important to increase patient survival.

With ADO, care must be taken in the removal of the bone marrow sequestration, as it is easy to create iatrogenic fractures due to bone density, which are difficult to repair once the bone is fractured.

During anesthesia, morbidity and mortality rates are still high in patients with osteopetrosis. The most common problem is intubation [13].

The literature supports surgery as the primary treatment for osteomyelitis, as antibiotics alone will not reach the compromised area [14].

## 4 Conclusion

In osteopetrosis, a precise and early diagnosis of the disease and the implementation of prophylactic treatment in the form of restorations and periodontal care is necessary to prevent the onset of infection.

Any oral surgery including extractions must be carefully planned and best performed in specialized centers.

The surgeon is often confronted with an intraoperative dilemma of how much to remove because there is a poor delineation between normal bone and necrotic bone.

Careful judgment is needed to balance the risk of leaving gross facial disfigurement and the risk of infection.

## Ethical approval

Written informed consent was obtained from the patient for publication of this case report and accompanying images. A copy of the written consent is available for review by the Editor-in-Chief of this journal on request.

## Sources of funding

The authors declared that this study has received no financial support.

## Author contribution

Raiteb Mohamed: Corresponding author writing the paper.

ELMRINI SANAA: writing the paper.

Faiçal Slimani: Correction of the paper.

## Registration of research studies

Name of the registry: researchregistry.

Unique Identifying number or registration ID.

Hyperlink to your specific registration (must be publicly accessible and will be checked).

## Guarantor

RAITEB MOHAMED.

## Consent

Written informed consent was obtained from the patient for publication of this case report and accompanying images. A copy of the written consent is available for review by the Editor-in-Chief of this journal on request.

## Declaration of competing interest

Authors of this article have no conflict or competing interests. All of the authors approved the final version of the manuscript.
